# Chemotaxis-driven disease-site targeting of therapeutic adult stem cells in dystrophic epidermolysis bullosa

**DOI:** 10.1186/s13287-016-0388-y

**Published:** 2016-08-27

**Authors:** Vitali Alexeev, Adele Donahue, Jouni Uitto, Olga Igoucheva

**Affiliations:** Department of Dermatology and Cutaneous Biology, Sidney Kimmel Medical College, Thomas Jefferson University, 233 South 10th Street, BLSB, Rm. 430, Philadelphia, PA 19107 USA

**Keywords:** Adipose-derived stem cells, Adult stem cells, Blistering skin, Chemokine receptors, Chemokines, Chemotaxis, Epidermolysis bullosa, Migration, Proteome analysis, Transplantation, Type VII collagen

## Abstract

**Background:**

Dystrophic epidermolysis bullosa (DEB), a rare genodermatosis, is characterized by the formation of intra-epidermal blistering and the development of chronic nonhealing skin wounds. Recently, attempts have been made to develop cell-based therapies for this currently intractable disorder. The molecular mechanisms that govern directional migration of the adult stem cells, allowing their efficient and controlled homing to the skin affected with DEB, are poorly understood. The key mechanism that regulates recruitment of leukocytes and progenitor stem cells to distal anatomical tissues affected with disease is chemotaxis, which depends on the signaling molecules, chemokines, and acts primarily as part of the host defense and repair mechanism.

**Methods:**

Comprehensive proteomic screening of chemokines in the blister fluids of DEB-affected mice was conducted to define the inflammatory and immune activities, thus providing potential to examine local biological mechanisms and define the protein signature within lesional skin as a potential marker of disease activity. Also, the therapeutic relevance of identified chemotactic pathways was investigated in vivo, providing a basis for future clinical investigations.

**Results:**

Assessment of blister fluid-derived chemokines showed a persistent presence of several chemotactic molecules, including CXCL1 + 2 and CXCL5. The majority of blister-originated chemotactic signals were associated with preferential recruitment of CD45^+^CXCR2^+^ and CD11b^+^CXCR2^+^ leukocytes. Systemic transplantation of an enriched CXCR2 population of mouse adipose-derived stem cells (mADSC) into DEB-affected mice demonstrated effective recruitment of cells to the blistering skin under the influence of blister-derived ligands and deposition of therapeutic type VII collagen.

**Conclusions:**

Collectively, these studies demonstrate that recruitment of mADSC into DEB skin is tightly controlled by disease-site chemotactic activities and suggest a potential mechanism for effective application of therapeutic stem cells for DEB.

## Background

Dystrophic epidermolysis bullosa (DEB) is the mechanobullous disorder characterized by complete lack or dysfunction of type VII collagen, resulting in separation of the dermal–epidermal junction (DEJ) below the lamina densa following minor or insignificant trauma to the skin [1]. During the past two decades, various therapeutic approaches have been proposed and tested in preclinical and clinical settings [2]; however, effective therapies allowing correction of this debilitating disorder remain to be developed.

In recent years, adult stem cell-based therapy was suggested as a potential approach to attenuate DEB progression. We and others have demonstrated that adult stem cells (ASC) of different origins (bone marrow, adipose tissue, blood vessels, and umbilical cord) produce a repertoire of extracellular matrix proteins, including type VII collagen, and a plethora of signaling molecules, such as growth factors and cytokines [3–8]. Also, we showed that intracutaneous transplantation of genetically normal ASC from bone marrow into DEB-affected mouse skin restores the DEJ via direct donation of the therapeutic type VII collagen [8]. These studies provided proof-of-concept data suggesting that adult stem cell-derived connective tissue proteins can participate directly in restoration of defective extracellular matrix and demonstrating the feasibility of systemic cellular therapy for DEB. In fact, data collected from ongoing clinical studies aimed at the application of whole bone marrow and bone marrow-derived mesenchymal stem cells (MSC) for DEB treatment showed that systemic allotransplantation accelerates wound healing, enhances angiogenesis, inhibits inflammation, and alleviates symptoms associated with the disease [9, 10]. However, despite encouraging results, these trials revealed that the majority of curative effects resulted from the secretion of various paracrine factors elicited by transplanted cells rather than from donation of the therapeutic protein to the affected cutaneous tissue [11].

Successful application of ASC for the correction of genodermatoses, including DEB, and treatment of chronic wounds which often accompany these disorders, mainly depends on the efficacy of stem cell homing to the damaged skin and is considered a major issue in stem cell-based therapy. The paucity and inefficient migration of ASC to the damaged skin have been documented in multiple wound healing studies [12, 13]. The causatives of the inefficient homing of ASC are still not defined but current data suggest that these deficiencies may arise, in part, due to poor extravasation of ASC from circulation into the cutaneous tissue. The molecular mechanisms regulating trafficking of the ASC to the skin, especially blistering skin, are poorly understood. Under physiological conditions, the key mechanism that regulates cell migration is chemotaxis, which depends on signaling molecules, chemokines. Being secreted from cells in damaged peripheral tissue, chemokines recruit chemokine receptor-expressing cells, such as leukocytes, and possibly stem cells as part of host defense and repair mechanisms. The crucial role of chemokines in directional migration of ASC to various tissues, such as the heart, lung, bone marrow, and intestine, has been demonstrated previously [14, 15]. Thus, knowledge of chemokines expressed by the targeted tissue and cognate chemokine receptors expressed on the surface of the targeted stem cells is a prerequisite for successful targeting of therapeutic ASC. The primary challenge is therefore to integrate our current knowledge of chemotaxis into a rational design of the guidance system to recruit ASC to the skin affected by certain genodermatoses, such as DEB.

Because ASC are naturally involved in the regeneration/repair of multiple tissues, expression of chemokine receptors in ASC from various sources has been studied widely. Collectively, these data indicate that ASC express a limited repertoire of functional chemokine receptors [6, 16, 17]. Moreover, responsiveness of ASC to only a few chemokines was tested experimentally [16, 18–22]. To the contrary, from our knowledge of chemotaxis in repair processes, very limited information is currently available regarding chemotactic signals in the skin affected by various forms of hereditary blistering disorders. Skin-derived chemokines have been mostly characterized in association with recruitment of leukocytes during acute and chronic inflammation [23]. Elevated levels of CXCL12 and CCL21 were reported in blister fluids following skin burns, lichen planus, and cutaneous lupus erythematosus, where inflammatory and immune responses were supported by the recruitment of CXCR4^+^ and CCR7^+^ leukocytes, respectively [24–26]. However, wide expression of CXCL12 in multiple organs does not provide desirable specificity of ASC migration leading to significant “dilution” of the therapeutic effect. Importantly, the CXCL12–CXCR4 chemotactic axis was also shown to be responsible for the entrapment of systemically administered ASC in lungs. For the same token, it was suggested that an endogenously high level of CCL21 in secondary lymphoid organs is responsible for the observed nonspecific distribution of systemically transplanted ASC [27]. Collectively, these observations strongly suggest the need for alternative chemotactic pathways to target ASC specifically to the skin.

Based on current knowledge of T-cell trafficking [23], it is plausible that systemically transplanted ASC can be recruited from circulation to the skin by the activation of specific receptors on the surface of transplanted stem cells, followed by transcutaneous migration mediated by stem cell receptors under the influence of the DEB skin-derived chemokines. In this study, we conducted proteomic screening of chemotactic molecules in the blister fluids of DEB-affected mice, the model for severe autosomal recessive DEB, to define the inflammatory and immune activities during the active disease state. Pair-wise comparison of identified chemokines in blister fluids and receptors on the surface of mouse adipose-derived stem cells (mADSC) allowed us to define potential chemotactic axes for targeting of the cells into the blistering skin. We investigated the therapeutic relevance of identified chemotactic pathways in vivo, providing a basis for future clinical investigations. Identified molecules provided insight into the mechanisms that govern directional migration and intracutaneous trafficking of systemically infused stem cells, thus permitting broad and effective application of the therapeutic cells for DEB treatment.

## Methods

### Mouse strains

Wild-type C57BL/6 mice were purchased from The Jackson Laboratory (Bar Harbor, ME, USA). Transgenic type VII collagen-deficient (*Col7a1*^*–/–*^) mice were generated in the Department of Dermatology and Cutaneous Biology, Thomas Jefferson University [28]. Transgenic type VII collagen-deficient hypomorphic (*Col7a1*^*f lNeo/f lNeo*^) mice were obtained from Freiburg Institute for Advanced Studies, Germany [29]. Both mouse models recapitulate the clinical, genetic, and ultrastructural features of human DEB. Targeted inactivation of the *Col7a1* gene generated a severely affected collagen VII knockout mouse (*Col7a1*^*–/–*^), which is born with extensive cutaneous blistering and rapid demise during the first 2 weeks of life due to complications arising from blistering in the skin, oral mucosa, and esophagus and imbalanced digestion by the gastrointestinal track. The second model, a collagen VII hypomorphic mouse (*Col7a1*^*f lNeo/f lNeo*^) which had 10 % of the normal collagen VII levels in the skin, developed all of the symptoms of severe RDEB but the presence of one-tenth of normal full-length type VII collagen confers a milder phenotype and better prognosis than that in *Col7a1* knockout mice.

### Blister fluid collection from DEB-affected mice

*Col7a1*^*–/–*^ and *Col7a1*^*f lNeo/f lNeo*^ mice are born with a blistering phenotype. Hemorrhagic blisters are readily developed on paws and other parts on the body (e.g., abdomen, armpit, neck). The blister fluids were collected by needle piercing with an attached syringe, cleared by centrifugation, and stored at –70 °C until testing.

### Chemokine antibody arrays

Proteome Profiler™ Mouse Chemokine Antibody Array (R&D Systems, Minneapolis, MN, USA) was employed to assay blister fluid samples derived from *Col7a1*^*–/–*^ and *Col7a1*^*f lNeo/f lNeo*^ mice, respectively. Twenty microliters of blister fluid was used to probe the chemokine antibody arrays according to the manufacturer’s instructions. Chemokine antibody array membranes were developed by standard enhanced chemiluminescence techniques as advised by the manufacturer. Acquisition of signals on mouse chemokine arrays was quantitatively determined using ScanAlize version 2.50 (Stanford University) and GEArray Expression Analysis Suite 2.0 software (SABiosciences, Frederick, MD, USA), which reads the images and matches them to the corresponding protein on the array. The net level of each protein was calculated by the mean of the individual spot intensity minus the mean of the background intensity. To provide normalization, the average level ratio of two principal genes was determined and introduced as a correction factor. Relative spot intensities are presented as mean ± SD. Microsoft Excel (Microsoft, Redmond, WA, USA) was utilized for statistical analysis.

### Isolation of mADSC and tissue culture conditions

mADSC were isolated from subcutaneous fat of wild-type C57 BL/6 J mice. Following collection, specimens were washed in PBS + 1 % Pen/Strep (Gibco, Grand Island, NY, USA) twice, minced into small pieces, and digested in collagenase solution (0.1 μg collagenase I (Sigma, St. Louis, MO, USA) in 1 ml PBS and bovine serum albumin (BSA)). To obtain a single cell suspension, the digested tissue was applied to a 30 μm mesh separation filter (Miltenyi Biotec, Auburn, CA, USA). PBS + 1 % BSA solution was added to the mesh to quench the enzyme and flush any remaining cells through the filter. The suspension was centrifuged and the pellet was resuspended in 1 ml of DMEM/F12 and Glutamax + 10 % FBS (Gibco). Cells were plated in DMEM/F12 and Glutamax + 10 % FBS (Invitrogen, Grand Island, NY, USA) and grown to confluence. The adherent cells (passage 0) underwent negative selection using magnetic beads (MACS; Miltenyi Biotec) to remove contaminating endothelial CD31^+^ and mononuclear CD45^+^ cells. Briefly, cells were released by trypsin and centrifuged at 300 × *g*. The pellet was suspended in 2 mM EDTA and 0.5 % BSA (MACS buffer), mixed with CD31^+^ and CD45^+^ tagged microbeads, and incubated at 4 °C for 15 minutes. After centrifugation, the cells were suspended in MACS buffer and passed through an LS Separation Column mounted in the QuadroMACS separator magnetic unit. The resultant CD31^–^CD45^–^ mouse cell population defined as mADSC was grown in standard DMEM/F12 and Glutamax + 10 % FBS media unless stated otherwise.

### Fluorescence-activated cell sorting analysis of mADSC

mADSC were grown until confluent, trypsinized, and pelleted by centrifugation at 200 × *g* for 5 minutes. For fluorescence-activated cell sorting (FACS) analysis, ~1.0 × 10^5^ cells were resuspended in 100 μl FACS buffer containing 1 % fetal bovine serum in PBS. For FACS analysis of surface receptors, each sample was incubated for 30 minutes at 4 °C with FITC-conjugated, Alexa488-conjugated, PerCP/Cy5.5-conjugated, PE-conjugated, or Alexa Fluor-647-conjugated antibodies against the surface markers CCR2, CCR3, CCR4, CCR5, CCR6, CCR7, CCR9, CCR10, CXCR1, CXCR2, CXCR3, CXCR4, CXCR6, CXCR5, CXCR6, and CXCR7 (eBioscience, San Diego, CA, USA) according to the manufacturer’s instructions. After incubation, the labeled cells were diluted with 2 ml of FACS buffer, pelleted and resuspended in 300 μl of FACS buffer. Generally, ~5 × 10^4^ cells were analyzed per sample using the Guava flow cytometer (BD Biosciences, San Jose, CA, USA). Results were analyzed using GuavaSoft 2.7 software (BD Biosciences).

### Generation and characterization of CXCR2-overexpressing mADSC

Full-length mouse Cxcr2 receptor with 3′ UTR was amplified from total mouse RNA via reverse transcription reaction using the Superscript II RT Kit (Invitrogen, Carlsbad, CA, USA) followed by PCR using PFU II high fidelity polymerase (Agilent Technologies, Santa Clara, CA, USA). Resultant cDNA was inserted into pEF2-TOPO vector. Integrity of the promoter and cDNA was verified by direct DNA sequencing. Minimally cultured mADSC (passages 1–2) were nucleofected with resultant plasmid (pEF1-mCxcr2) using Lonza nucleofection reaction (T-27 program, nucleofection kit V; Lonza, Cologne, Germany). Further, a pool of CXCR2-expressing cells was selected with Blasticidin (0.5 mg/ml; Invitrogen) for 10 days. Expression of CXCR2 in selected cells was confirmed by FACS and indirect immunofluorescence analyses. Surface expression of CXCR2 was determined by FACS using PE-conjugated antibodies as already described. For indirect immunofluorescence, CXCR2 immunocomplexes were detected with Alexa-Fluor^488^-conjugated secondary antibodies (Invitrogen). Nuclei were counterstained with 4′,6-diamidino-2-phenyl indol (DAPI; Sigma). Immunofluorescent images were obtained on a Nikon TS100F fluorescent microscope (Nikon, Melville, NY, USA).

### Labeling CXCR2^+^ mADSC and transplantation into Col7a1^–/–^ mice

All animal procedures were performed in accordance with the *Guide for the Care and Use of Laboratory Animals* (National Institutes of Health publication no. 86-23) and approved by the Institutional Animal Care and Use Committee of the Thomas Jefferson University. For all transplantation studies, mADSC (passages 2–4) isolated from wild-type C57BL/6 J were labeled with a red lipophilic tracer DiOC18 (Molecular Probes, Grand Island, NY, USA) as we described previously [6]. For systemic transplantation, the 1–2-day-old *Col7a1*^*–/–*^ neonates (*n* = 5/time point) received 0.6 × 10^6^–0.8 × 10^6^ cells in 10–15 μl PBS per mouse intraperitoneally. For direct viewing, transplanted cells were detected using an IVIS live-imaging system (IVIS Lumina XR; Caliper LifeSciences, Alameda, CA, USA). To avoid detection of autofluorescence, the exposure time of all imaged animals was minimized to a maximum of 1 second.

### Histological and immunofluorescent analyses

Collected skin tissue was embedded into OCT compound (VWR, Radnor, PA, USA), frozen, and cryosectioned at a thickness of 7 μm. For histological analysis, sections were stained with H&E using a standard protocol. For direct viewing of transplanted mADSC, sections were fixed with 10 % formalin, stained with DAPI (Sigma) and evaluated using fluorescence microscopy. For indirect immunofluorescent analysis, cross-sections were stained with the following primary antibodies: goat anti-mouse CD45 polyclonal (BD Bioscience), rat anti-mouse CD11b monoclonal (BD Bioscience), rabbit anti-CXCR2 polyclonal (Bioss, Woburn, MA, USA), mouse anti-FLuc monoclonal (Abcam, Cambridge, MA, USA), rabbit anti-type VII collagen polyclonal (Millipore, Billerica, MA, USA), and goat anti-type IV collagen polyclonal (Millipore). Immunocomplexes were detected with AlexaFluor^488^-labeled and AlexaFluor^594^-labeled secondary antibodies (Invitrogen), respectively. Nuclei were counterstained with DAPI. Immunofluorescent images were obtained on a Nikon TS100F fluorescent microscope (Nikon). For analysis of the type VII collagen deposition, stained sections were analyzed using AutoQuant imaging software (AutoQuant Imaging Inc., Troy, NY, USA).The average value of the basement membrane zone (BMZ)-accolated fluorescence was calculated based on the ratio of the average value of the total fluorescence of wild-type skin (designated as 100 %) and the relative percentage of fluorescence in the skin of transplanted mice. Differences in fluorescence intensities were assessed using a Student’s *t* test, with *p* < 0.01 considered significant in all tests.

### Statistical analysis

All data are expressed as mean ± SD. Statistical significance was assessed using two-tailed type 2 Student’s *t* test, where differences with *p* < 0.05 were considered significant.

## Results

### Proteomic screens of chemotactic molecules in DEB-affected mouse skin

**Fig. 1:**
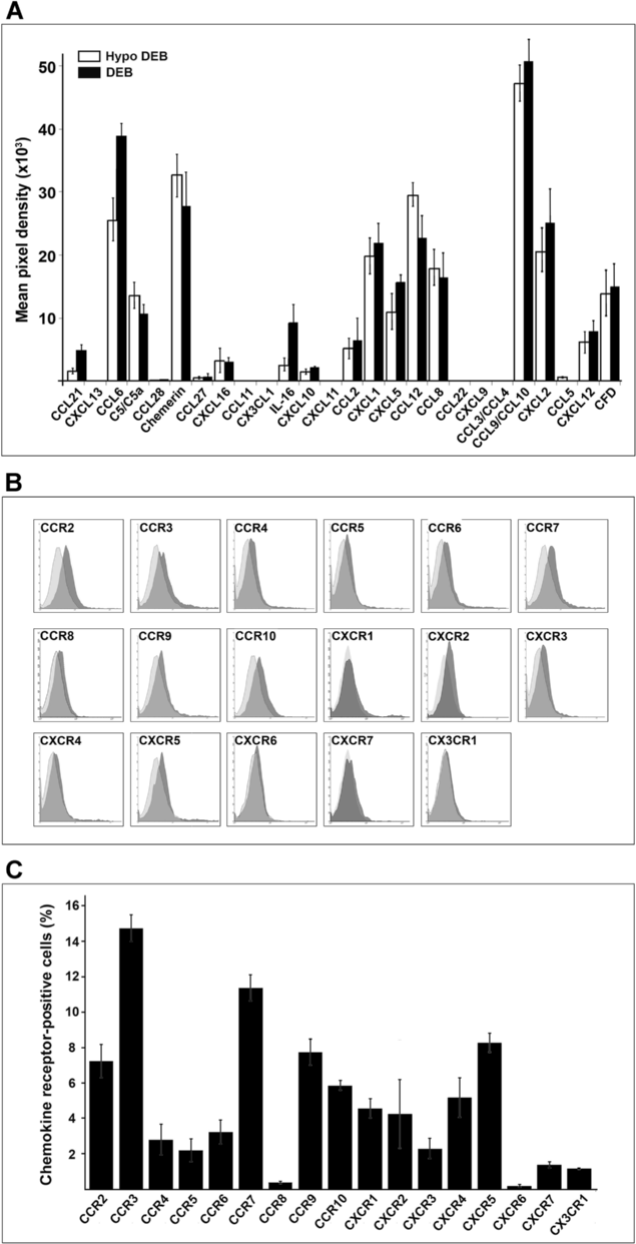
Analysis of chemokines in DEB-associated blister fluids and chemokine receptors on the surface of mADSC. **a** Chemokine profile in blister fluids of *Col7a1* ^*–/–*^ (*DEB*) and *Col7a1* ^*f lNeo/f lNeo*^ (*Hypo DEB*) mice conducted using Proteome Profiler™ Mouse Chemokine Antibody Array. Blister fluids were collected from blister-affected skin (paw, armpit, neck, and abdomen) of newborn mice. Spot intensities were normalized based on background levels and positive control signals. Data shown as mean ± SD from three independent arrays (*p* > 0.05). **b** Surface expression of chemokine receptors in primary mADSC on early passage (passages 2–3) detected by flow cytometry. *Light gray curves*, control antibody signal; *dark shaded curves*, specific antibody signal. Histograms are representative examples of chemokine receptor expression profiles obtained from five independent mADSC preparations. **c** Quantitation of flow cytometry results for expression of the chemokine receptors in primary mADSC. Data shown as mean ± SD of cell percentage. *DEB* dystrophic epidermolysis bullosa

The chemokine expression profiles in blister fluids of *Col7a1*^*–/–*^ knockout mice and *Col7a1*^*f lNeo/f lNeo*^ hypomorphic mice were studied using Proteome Profiler™ Mouse Chemokine Antibody Array allowing the detection and quantification of 35 distinct chemotactic molecules. The chemokine arrays were probed using blister samples collected from blistering skin of mice. Proteomic screens of blister fluids collected from *Col7a1*^*–/–*^ (*n* = 5) and *Col7a1*^*f lNeo/f lNeo*^ (*n* = 5) mice showed consistently high levels of several chemotactic molecules, including CCL6, chemerin, CCL8, CCL9/CCL10, CCL12, CXCL1, CXCLl2, CXCL5, and CDF (Fig. 1a).

The analysis of biological function of identified chemokines suggested potential inflammatory infiltrate in blistering skin. For example, CCL6 has only been identified in rodents and is a potent chemoattractant of macrophages as well as B cells, CD4^+^ lymphocytes, and eosinophils. In mice, CCL6 is expressed in cells from neutrophil and macrophage lineages, and can be induced under conditions suitable for myeloid cell differentiation. The cell surface receptor for CCL6 is believed to be the chemokine receptor CCR1. Chemerin, also known as retinoic acid receptor responder protein 2 (RARRES2), is a chemoattractant protein that acts as a ligand for the G protein-coupled receptor CMKLR1, also known as ChemR23. Chemerin was found to stimulate chemotaxis of dendritic cells and macrophages to the site of inflammation. CCL8, also known as monocyte chemoattractant protein 2 (MCP-2), is chemotactic for and activates many different immune cells, including mast cells, eosinophils, and basophils as well as monocytes, T cells, and NK cells that are involved in the inflammatory response. CCL8 elicits its effects by binding to several different cell surface receptors, including CCR1, CCR2B, and CCR5. CCL9/CCL10, also called macrophage inflammatory protein-1 gamma (MIP-1γ), macrophage inflammatory protein-related protein-2 (MRP-2), or CCF18, in rodents attracts dendritic cells that possess the cell surface molecule CD11b and the chemokine receptor CCR1. CCL9 is constitutively expressed in macrophages and myeloid cells. CCL12, also known as monocyte chemotactic protein 5 (MCP-5) or MCP-1-related chemokine, specifically attracts eosinophils, monocytes, and lymphocytes. Its expression can be hugely induced in macrophages. CCL12 is a ligand for CCR2. CXCL1/2/3, also known as growth-regulated oncogene (GRO), can bind with high affinity to the IL-8 receptor type B and is a very potent neutrophil attractant and activator. CXCL5, also known as epithelial-derived neutrophil-activating peptide 78 (ENA-78), is produced following stimulation of cells with the inflammatory cytokines interleukin-1 or tumor necrosis factor alpha. CXCL5 is well known to have chemotactic and activating functions on neutrophils, mainly during acute inflammatory responses. This chemokine stimulates the chemotaxis of neutrophils possessing angiogenic properties and has been implicated in connective tissue remodeling. The cell surface receptor for CXCL5 is the chemokine receptor CXCR2.

Collectively, all identified molecules are prominent chemotactic factors that activate and attract a variety of inflammatory cells to the sites of inflammation produced by tissue injury or disease. Also, the identified chemokine profiles provided several potential lead-chemotactic gradients, including CCL6–CCR1, CCL8–CCR1/CCR5, CCL9/10–CCR1, CCL12–CCR2, CXCL1/2–CXCR2, and CXCL5–CXCR2, for efficient recruitment of therapeutic stem cells into the skin affected with blisters.

### Expression profile of chemokine receptors in mADSC

The presence of active chemokine receptors on mADSC was examined in order to determine potential migratory ability upon in-vivo transplantation into mice. A series of mADSC preparations were generated from subcutaneous fat of C57BL6J mice (*n* = 5), representing actively dividing cultures at early passage (passage 2). Cell surface expression for receptors on the primary mADSC was characterized by FACS analysis using fluorescently-labeled receptor-specific antibodies against a panel of available mouse chemokine receptors. The analysis showed that the mADSC express the limited repertoire of functional receptors, consistent with the published profiles from mouse adipose tissue (Fig. 1b). The overall level of receptor expression showed consistent reproducibility in all tested mADSC cultures.

The expression of chemokine receptors appeared heterogeneous. A small percentage of cells was positive for CCR5 (4.5 ± 1.1 %), CCR6 (4.1 ± 3.8 %), CCR8 (0.9 ± 2.8 %), CXCR1 (3.0 ± 2.4 %), CXCR2 (2.5 ± 3.2 %), CXCR3 (4.2 ± 3.1 %), CXCR6 (0.3 ± 3.5 %), CXCR7 (2.2 ± 1.1 %), and CX3CR1 (2.3 ± 3.1 %). A higher percentage of cells was positive for CCR2 (7.8 ± 4.1 %), CCR3 (14.0 ± 4.3 %), CCR4 (6.5 ± 2.1 %), CCR7 (10.9 ± 5.2 %), CCR9 (8.2 ± 2.9 %), CCR10 (6.1 ± 4.1 %), CXCR4 (7.0 ± 1.7 %), and CXCR5 (8.7 ± 3.5 %) (Fig. 1c). Primary mADSC culture thus showed a heterogeneous phenotype, in which functional chemokine receptors are not expressed uniformly and only limited yet uncharacterized subsets of ADSC express specific receptors at relatively low levels, suggesting that only a small fraction of cells is available for effective recruitment of transplanted cells into the skin.

### Chemotactic recruitment of mADSC to DEB-affected blistering and nonblistering skin

**Fig. 2:**
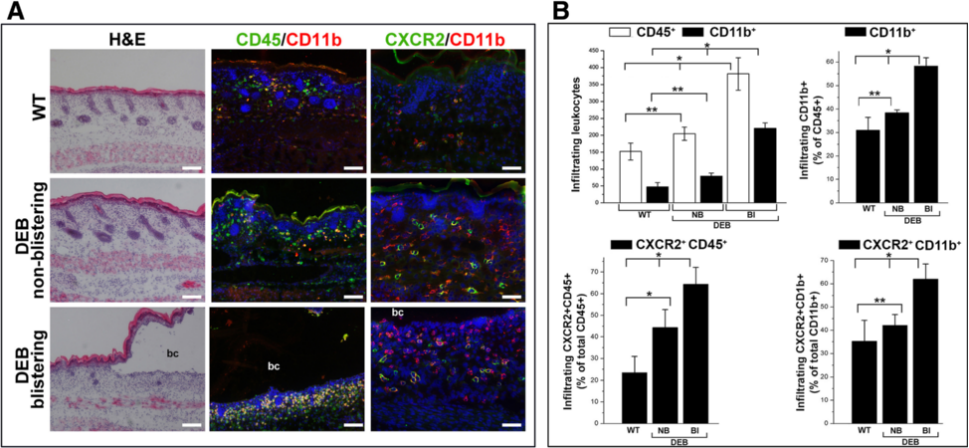
Analysis of wild-type and DEB-affected skin infiltration with leukocytes. **a** H&E staining and indirect immunofluorescent analysis of skin infiltration with leukocytes. Detected antigens are shown in respective colors above the panels (CD45, *green*; CXCR2, *green*; CD11b, *red*; colocalization of CD45 or CXCR2 and CD11b, *yellow*). Nuclei were counterstained with DAPI (*blue*). *bc* blister cavity. *Scale bar* = 100 μm. **b** Quantitation of the skin-infiltrating cells. Data presented as an average number or percentage of cells (as indicated on the *Y* axis) per microscopic field ± SD. *p <0.05. **p > 0.05. Data collected from at least 15 independent microscopic fields. CD-positive cell types are shown above the columns. *H&E* hematoxylin and eosin, *WT* wild-type skin, *NB* nonblistering, *DEB* dystrophic epidermolysis bullosa, *Bl* blistering DEB-affected skin

Analysis of the blistering and nonblistering skin in 1–2-day-old *Col7a1*^*–/–*^ and wild-type neonatal mice demonstrated a significant infiltration of the DEB-affected skin with leukocytes (Fig. 2a). Infiltrates were detected at both ventral skin and the skin of the extremities, the most common sites of blister formation in DEB mice. However, it was particularly noticeable in the blistering skin where leukocyte infiltrate extended into the blister cavity (Fig. 2a). Immunophenotyping analysis showed that about 30 % of CD45^+^ leukocytes in the wild-type skin were represented by the CD11b^+^ cells. The percentage of CD11b^+^ cells in the DEB-affected nonblistering skin was slightly elevated whereas in blistering skin it was two times higher (Fig. 2a, b).

Considering elevated levels of CXCR2 ligands, CXCL1, CXCL2, and CXCL5, in blister fluids, skin-infiltrating CXCR2^+^ cells were further quantitated. As expected, in normal mouse skin about 24 % of CD45^+^ leukocytes expressed CXCR2. In DEB-affected nonblistering skin, this percentage was higher, approaching 45 %. In the blistering DEB skin, more than 60 % of all leukocytes were CXCR2^+^. The prevalence of CD11b^+^ cells in cutaneous infiltrates and the high percentage of CXCR2^+^CD11b^+^ cells in the blistering skin suggest that higher levels of the CXCR2 ligands, CXCL1, CXCL2, and CXCL5, in blister fluids may direct intracutaneous migration of the CXCR2^+^ leukocytes. These findings also suggest that CXCR2 could mediate homing of the systemically infused therapeutic type VII collagen-producing mADSC to the blistering and nonblistering DEB-affected skin.

### Cxcr2-mediated homing of the mADSC to the DEB-affected skin

**Fig. 3:**
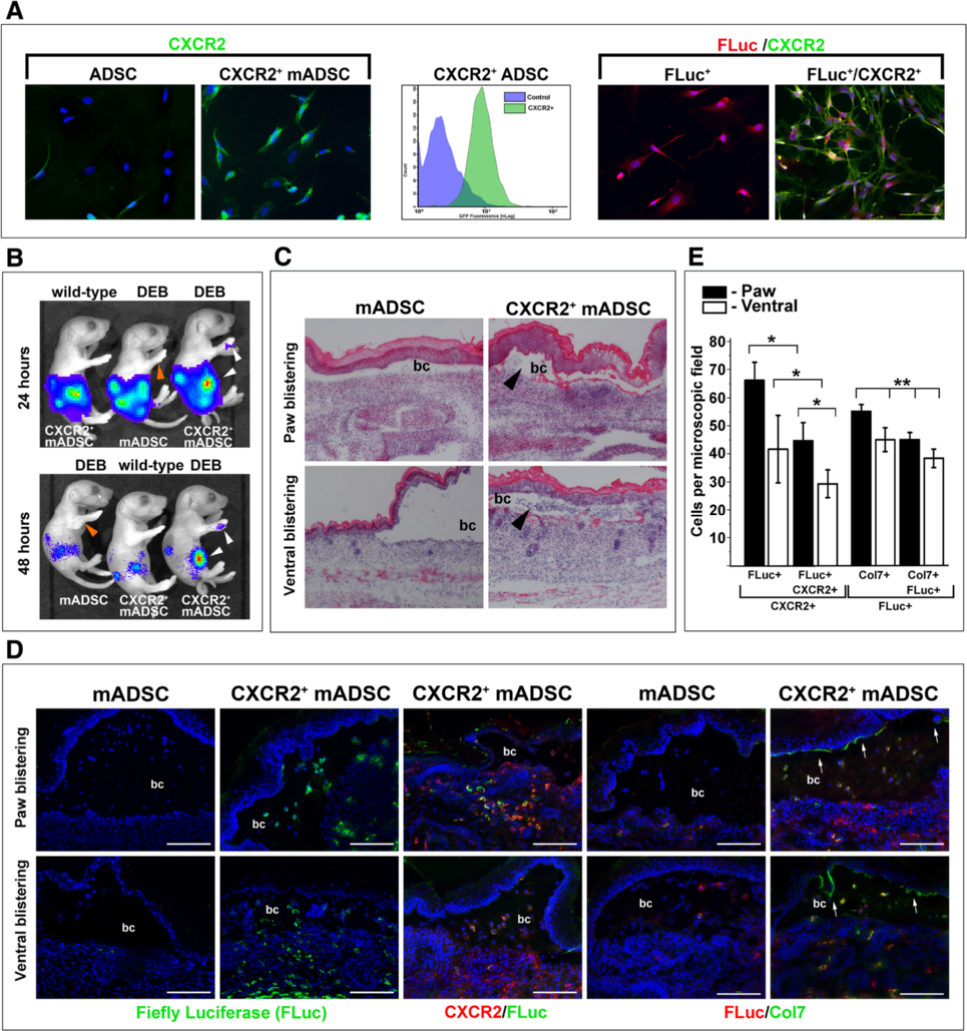
Systemic transplantation of genetically engineered CXCR2^+^-mADSC into DEB-affected newborn mice. **a** Characterization of CXCR2 expression in primary mADSC and cells engineered to overexpress FLuc (FLuc^+^-mADSC) and FLuc and *Cxcr2* genes (FLuc^+^CXCR2^+^-mADSC), respectively, by FACS and indirect immunofluorescent detection. Analyses were performed using gene-specific antibodies. (Fluc, *red*; CXCR2, *green*; colocalization of FLuc and CXCR2, *yellow*; nuclei were counterstained with DAPI). **b** In-vivo imaging of systemically administered FLuc^+^CXCR2^+^-mADSC and native FLuc^+^-mADSC into newborn *Col7a1* ^*–/–*^ and wild-type mice, respectively, 24 and 48 hours after transplantation (as indicated on the *left*). *White and orange arrows* point to the blister location. Type of transplanted cells indicated below the image of a representative mouse. **c** Representative micrographs of H&E-stained sections of the skin collected from blistering sites (ventral skin and paws) of FLuc^+^-mADSC and FLuc^+^CXCR2^+^-mADSC transplanted animals, respectively. *Black arrowheads* point to infiltrating cells. *bc* blister cavity. **d** Indirect immunofluorescent analysis of mADSC recruitment to blistering sites (indicated to the *left* of the panels). Type of mADSC used for transplantation indicated above the panels. Detected antigens, FLuc, CXCR2, and Col7, are shown below the panels in corresponding colors. *Scale bar* = 100 μm. *bc* blister cavity, *white arrows* point to the stretches of the BMZ-associated type VI collagen. **e** Quantitation of cell recruitment to blistering sites. *Y* axis represents the percentage of positive cells per microscopic field. The FLuc^+^, CXCR2^+^, and Col7^+^ cells are shown below the columns. Data shown as mean ± SD. *p <0.05. **p > 0.05. *DEB* dystrophic epidermolysis bullosa, *mADSC* mouse adipose-derived stem cells

FASC analysis showed the presence of a small percentage of CXCR2-expressing mADSC (2.5 ± 3.2 %) in the total pool of minimally cultured cells, suggesting that homing of systemically administered mADSC to the skin could be enhanced via the use of cells uniformly expressing CXCR2 receptor. Thus, we generated a stable cell line of mADSC expressing firefly luciferase (FLuc^+^-mADSC) and a derivative line coexpressing FLuc and mouse *Cxcr2* genes (FLuc^+^CXCR2^+^-mADSC), respectively (Fig. 3a). One cohort of wild-type newborn mice (*n* = 3 per cohort) and two cohorts of *Col7a1*^*–/–*^ newborn animals (*n* = 3 per cohort) received an intraperitoneal (IP) injection of FLuc^+^CXCR2^+^-mADSC and parental FLuc^+^-mADSC (5 × 10^5^ cells per injection), respectively. Twenty-four hours after injection, FLuc signals were assessed using the AVIS live-imaging system. As expected, FLuc activity was found associated with the intraperitoneal cavity and spleen in all recipient mice (Fig. 3b). Importantly, FLuc activity signals were readily detectable in the blister sites located on the paws and the ventral skin of mice treated with FLuc^+^CXCR2^+^-mADSC but not in animals receiving FLuc^+^-mADSC transplant. An additional 24 hours resulted in the accumulation of the FLuc^+^CXCR2^+^-mADSC to the blister sites (Fig. 3b). H&E staining revealed an increased number of the skin-infiltrating cells at the blistering skin of mice receiving FLuc^+^CXCR2^+^-mADSC. Infiltrate was mostly noticeable at both ventral-associated and paw-associated blistering sites where penetration of infiltrating cells was readily seen in the blister cavity (Fig. 3c). Indirect immunofluorescent detection of the FLuc confirmed the presence of the transplanted FLuc^+^CXCR2^++^-mADSC in the blistering skin, whereas unselected FLuc^+^-mADSC were rarely detected at these sites (Fig. 3d). Double-immunostaining showed that 40–60 % of CXCR2^+^ cells express FLuc in ventral and paw-blistering skin (Fig. 3c, d), suggesting that more than 50 % of skin-recruited cells are represented by the transplanted FLuc^+^CXCR2^+^-mADSC. Further analysis of FLuc^+^CXCR2^+^-mADSC transplant showed the persistent presence of type VII collagen and FLuc double-positive cells in the upper dermis as well as within the blister cavity. These double-positive cells represented about 40–50 % of all transplanted FLuc^+^ cells (Fig. 3d, e). Significantly, short stretches of type VII collagen protein were also detected at the basal layer of the epidermis (Fig. 3d). Contrary, type VII collagen protein was undetectable in the skin of mice receiving unselected FLuc^+^-mADSC transplant. Collectively, these studies demonstrated that transplantation of mADSC uniformly expressing CXCR2^+^ receptor, unlike unselected counterparts, results in efficient recruitment of stem cells from circulation directly to the blistering skin, where cells exert their therapeutic function by production of collagen type VII protein in the proximity of the cutaneous BMZ.

### Enrichment of the Cxcr2^+^-mADSC population for skin homing

The obtained data demonstrate that genetically engineered CXCR2^+^-expressing mADSC can be effectively targeted to the blistering skin due to engagement and activation of CXCR2 by the DEB skin-producing ligands, CXCL1, CXCL2, and CXCL5. However, to make this approach clinically relevant, more generalized stem cell selection strategy to enrich the cells expressing lead receptors is required. To obtain a CXCR2^+^-enriched population, antibody-mediated positive selection of mADSC on paramagnetic microbeads with consequent removal of the beads via cleavage of the antibody-bead linker using CEllection biotin binder kit was tested. However, this approach did not produce a desirable number of viable mADSC (data not shown).

**Fig. 4:**
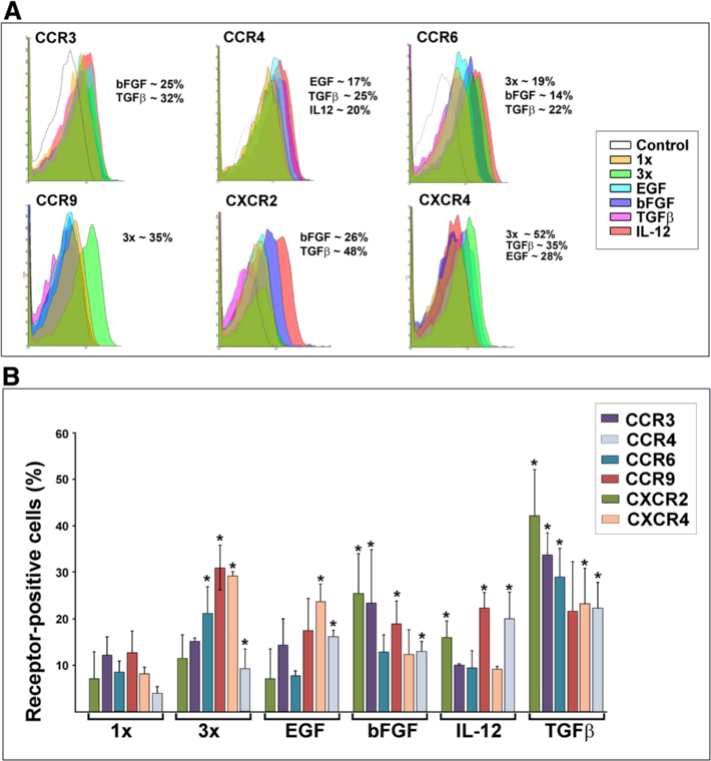
Characterization of cell surface chemokine receptors on mADSC after treatment with selected growth factors and cytokines. **a** FACS-based quantitation of the chemokine receptor-positive populations of mADSC under different culture conditions. Selected chemokine receptors identified on the surface of the cells indicated above the profiles, treatment conditions indicated on the panel: *1×* confluent culture, *3×* high-density culture. Treatments that increase populations of chemokine receptor-positive cells are indicated to the right of the profiles. **b** Percentage of receptor-positive cells in confluent cultures. Treatment conditions are shown to the right of the graphs. Data shown as mean ± SD from three independent experiments. *p < 0.05 compared with control (1×) cells

Previously, we observed that tissue culture conditions can significantly affect secretion of chemokines and chemokine receptors in bone marrow-derived MSC. In fact, assessment of chemokine receptors on the surface of freshly isolated and minimally cultured cells demonstrated expression of multiple receptors on rather small populations of the cells. Further analysis of the cell surface expression of CXCR2^+^ and other potential epithelia-targeting receptors on mADSC cultured at different densities and in the presence/absence of selected growth factors/cytokines was thus tested. FACS-based assessment showed that when cell proliferation was repressed using a contact inhibition approach by plating high-density culture exceeding confluency three times and continued cultured for additional 2 days, a substantial induction of several chemokine receptors was detected (Fig. 4a). Exposure of dense cultures to granulocyte macrophage colony-stimulating factor (GM-CSF; 100 ng/ml) did not alter expression of chemokine receptors (data not shown). However, culturing of cells with epidermal growth factor (EGF; 20 ng/ml) for 48 hours boosted cell surface expression of CCR4 and CXCR4, whereas treatment with basic fibroblast growth factor (bFGF; 25 ng/ml) induced CCR3, CCR4, CCR6, and CXCR2 (Fig. 4b). Interleukin-12 (IL-12; 25 ng/ml) preferentially induced CCR3, CCR4, CCR9, and CXCR2. Interestingly, transforming growth factor beta (TGF-β; 10 ng/ml) affected cell surface expression of all examined receptors with predominant induction of CCR3, CCR6, and CXCR2. Averagely, exposure of cells to TGF-β led to the enrichment of the CXCR2^+^-mADSC population ranging from 40 to 55 %. Considering the abundance of CXCR2 ligands in blister fluids, expression of CCR3 ligands (CCL11 and CCL24) in the skin, and expression of CCR4 ligands (CCL17 and CCL22) in dermal microvasculature, treatment of cells with TGF-β was selected as a stimulatory factor for CXCR2^+^-mADSC enrichment.

### Transplantation of the CXCR2-enriched mADSC into DEB-affected mice

**Fig. 5:**
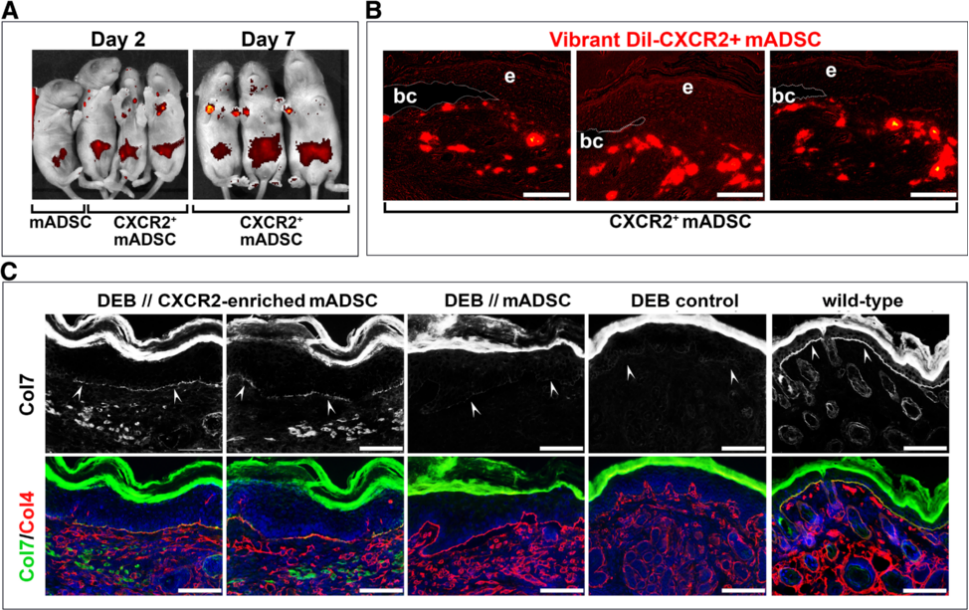
Transplantation of CXCR2-enriched mADSC into DEB-affected mice. **a** In-vivo live imaging of neonatal DEB mice and 2 and 7 days after IP transplantation of the DiI-labeled native and CXCR2^+^-mADSC (*red*), respectively. **b** Direct fluorescent detection of the DiI-mADSC in the blistering skin of the recipient mice at 7 days after transplantation. Blister cavity (*bc*) outlined by *dotted line*. **c** Indirect immunofluorescent detection of type VII and type IV collagens in the skin of mADSC-treated and control mice (indicated above the panels). Detected antigens (in corresponding color) are shown to the left of the panels. *Upper row*, monochrome images of the sections stained with anti-type VII collagen antibodies. *White arrowheads* point to the BMZ. *Blue*, DAPI nuclear staining. *Scale bar* = 100 μm. *DEB* dystrophic epidermolysis bullosa, *mADSC* mouse adipose-derived stem cells

To see whether the selected CXCR2^+^-mADSC provide an ADSC population with a functional receptor capable of directing extravasation at the blistering skin, unselected and CXCR2^+^-enriched mADSC were delivered via IP injection (1 × 10^6^ cells/injection/animal) into newborn 1-day-old *Col7a1*^*–/–*^ mice (*n* = 3), respectively. For transplantation of CXCR2^+^-mADSC, cells on early passage (passages 2–3) were plated in subconfluent density and cultured in the presence of TGF-β for 48 hours. To visualize the recruitment of cells to the blistering skin along natural CXCL1 + 2 and CXCL5 chemotactic axes, cells were labeled with Vibrant DiI red fluorescent tracker. As expected, during the first 24 hours transplanted cells were detected mostly at the abdomen, the site of injection, in both transplanted groups (Fig. 5a). However, DiI signals were readily detectable at blistering sites on the chest and neck of the mice receiving CXCR2^+^-mADSC. *Col7a1*^*–/–*^ recipients receiving unselected mADSC transplants demised shortly after transplantation on days 3 and 4. Analysis of the blistering skin of mice receiving unselected mADSC did not show any appreciable recruitment of mADSC to the blistering skin. In contrast, all recipients transplanted with CXCR2^+^-mADSC survived for at least 1 week without obvious signs of deterioration, although animals started to show a runted phenotype. At day 7, migration of CXCR2^+^-mADSC from the intraperitoneal cavity to the blistering paws and neck was observed as judged by accumulation of DiI fluorescent signals. Direct immunofluorescent evaluation of the blistering skin sections confirmed the presence of DiI^+^CXCR2^+^ cells detectable in the mid and upper dermis and in close proximity to the blister cavity (Fig. 5b). Indirect immunofluorescent analysis revealed type VII collagen-positive transplanted cells just below the epidermis with few cells detectable at the basal layer. Double-immunostaining with antibodies specific for type IV collagen showed a clear colocalization of both proteins at the BMZ (Fig. 5c), suggesting functionality of the donated protein and partial restoration of the DEJ. Quantitation of the collagen VII-associated fluorescence by measuring integrated density at the DEJ showed that transplantation of the CXCR2^+^-mADSC led to an increase of the type VII collagen corresponding to 45 % of the wild-type level with the mean gray value corresponding to 65 % of the wild-type skin. Collectively, the in-vivo studies demonstrated that an enriched population of CXCR2^+^-mADSC can efficiently migrate toward the blistering skin characterized by the elevated levels of CXCR2 ligands and donate functional therapeutic type protein into the BMZ of DEB-affected mice.

## Discussion

Several clinical studies were conducted to evaluate the utility of the ASC in alleviating DEB-associated symptoms [9, 10, 30, 31]. Although some degree of success was reported, there is a growing body of evidence that the observed therapeutic outcome was mainly associated with the transplanted cell-derived paracrine effects rather than a direct contribution of the cells to the restoration of the BMZ integrity [10]. One of the potential pitfalls in achieving desirable therapeutic effects could be associated with the poor migration and homing of the systemically transplanted therapeutic cells to the DEB skin affected with blisters.

Understanding the mechanisms regulating migration of systemically transplanted ASC is crucial to the success of any clinical strategy. Here we report a detailed study to address the chemotactic responsiveness of mADSC to natural chemotactic gradients in mice affected with severe DEB. To identify key players responsible for disease-targeting of systemically infused cells to the blistering skin, chemotactic signatures of the DEB-associated blister fluids were investigated by employing proteome profile screens. The content of chemotactic molecules in blister fluids showed consistently high levels of several chemokines, including CCL6, CCL8, CCL9/10, CCL12, CXCL1, CXCL2, and CXCL5. All identified molecules are prominent chemotactic factors that activate and attract various inflammatory cells, including lymphocytes, macrophages, neutrophils, and granulocytes. These data directly suggest that the presence of elevated levels of identified chemokines can provide favorable chemotactic axes (CCL8–CCR1, CCL12–CCR2, CXCL1 + 2–CXCR2, and CXCL5–CXCR2) for efficient recruitment of the therapeutic stem cells from the systemic compartment to the DEB-affected cutaneous tissue. From a clinical perspective, the CCL6–CCR1 and CCL9/10–CCR1 axes can be excluded because the ligands have only been described in rodents.

Because several of the upregulated chemokines (CXCL1, CXCL2, and CXCL5) interact with CXCR2 chemokine receptor, natural recruitment of the CXCR2-positive leukocytes to the blistering and nonblistering skin of the DEB-affected mice was further assessed. Analysis demonstrated that up to 40 % and 70 % of all leukocytes in nonblistering and blistering skin, respectively, express CXCR2 on the cell surface, suggesting that CXCR2 ligands play an important role in recruiting CXCR2^+^ leukocytes to the blistering sites. Systemic transplantation of the mADSC engineered to uniformly express CXCR2 confirmed this notion and demonstrated preferential recruitment of the CXCR2^+^-mADSC cells to blistering skin when compared with a native cell population. This observation is in good agreement with prior studies when chemotactic molecules were used to target stem cells to specific organs [32, 33]. Recently, we showed that MSC overexpressing CCR4^+^ and CCR10^+^ could be recruited to epithelial tissue toward an ectopic gradient of endothelial cell-derived CCL17 and epithelia-derived CCL27 axes and take part in regeneration/repair of the skin [7]. Our findings supported the idea that CCR4 engagement is essential for binding to the endothelium and extravasation, whereas activation of CCR10 is crucial for the directional migration of the cells within the collagenous matrix. Also, these data suggested distinct yet synergistic CCR4-dependent and CCR10-dependent mechanisms of stem cell recruitment to the skin. There has been much work to investigate the role of the CXCL12–CXCR4 axis to target MSC to diverse tissues, including ischemic heart, brain, skeletal muscle, kidney, liver, and skin [34]. To date, this is the most studied axis in MSC homing to wounds [24, 35]. Despite this interest, the majority of these studies investigated cell homing to sites of burn injury or radiation. In a limited number of investigations in cutaneous wounds, it was reported that skin grafts can recruit bone marrow-derived MSC through stromal derived factor-1α (SDF-1α, also known as CXCL12)/CXCR4 interaction to enhance tissue regeneration. Data suggested that during a skin graft hypoxic damage to the skin results in release of a soluble SDF-1α, which in turn recruits CXCR4^+^ MSC from circulation to the graft. However, considering wide expression of CXCL12 in multiple organs and uninjured tissues, reduced targeting specificity can significantly dilute the therapeutic effect. Furthermore, HMGB1 released from the detached or blistered DEB epithelia was suggested to be a factor in mobilizing Lin^–^/PDGFRα^+^ bone marrow cells into circulation and recruitment of cells to the epidermis and dermis of grafted Col7-deficient skin on the back of GFP-BMT mice and acceleration of skin regeneration [36]. Although an interesting experimental concept, recruitment of PDGFRα^+^ cells by HMGB1 does not provide any homing specificity and strictly depends on the apoptotic and necrotic status of the tissue. Also, there are no in-vivo data supporting systemic recruitment of PDGFRα^+^ cells into the skin of DEB mice. Based on our comprehensive whole genome oligo microarray profiles, the primary ADSC naturally express PDGFRα^+^ [6]. Moreover, analysis of blister fluids from patients affected with DEB showed an extremely low level of PDGFRα^+^ ligand, PDGF-BB (data not yet published). However, as shown in this study and our prior work [7], transplantation of primary heterogeneous stem cells failed to provide any appreciable recruitment of cells into the blistering and nonblistering skin, suggesting that PDGFRα^+^ alone is not sufficient to drive effective migration of cells from circulation into the skin. Taken together, high levels of CXCL1, CXCL2, and CXCL5 ligands observed in DEB-associated blister fluids can provide stronger and more specific chemotactic attraction of systemically administered cells expressing cognate CXCR2 receptor on its surface to the DEB-affected cutaneous tissue.

Generation of a stable mADSC cell line with engineered CXCR allowed achieving effective homing of systemically infused cells to blistering skin; however, genetic manipulation with cells may greatly complicate clinical application. To develop a strategy aimed at isolation and enrichment of the skin-homing ADSC, two approaches were considered: antibody-based separation of CXCR2^+^ cells on magnetic beads; and alteration of cell culture protocols. The former approach provided a good yield of freshly isolated cells after selection on paramagnetic microbeads but failed to produce sufficient number of viable cells following enzymatic cleavage of cells from the beads. The latter strategy, however, demonstrated that transient changes in cell culture condition may substantially enrich a population of cells expressing desired chemokine receptor. In fact, dense cultures produced induction of several chemokine receptors, including CCR3, CCR4, CCR6, CCR9, CXCR2, and CXCR4. Moreover, exposure of cells to TGF-β significantly increased population of cells expressing CXCR2. TGF-β, alternatively, resulted in induction of other potential skin-homing receptors, including CCR4 and CCR6, whereas incubation with bFGF altered CCR3 and CXCR2 expression. The presented data thus demonstrate that expression of lead receptors on mADSC could be altered by temporary changes in tissue culture conditions and suggest that the stem cells with disease-organ-specific homing capabilities could be generated, avoiding time-consuming, labor-retaining, and expensive selection protocols. At present, very limited information is available regarding the effect of various growth factors and cytokines on ADSC-specific cell surface receptor composition. The majority of studies are focused on optimization of stem cell proliferation, survival, and differentiation. In fact, TGF-β, PDGF, and FGF were shown to play an important role in proliferation of ADSC [37]. Currently, the focus of stem cell biology is primarily shifted on the trophic effects elicited by stem cells. Growing data suggest that therapeutic benefit may not be restricted to the repair function of cells alone, but also due to their transient paracrine actions. In fact, current research suggests that stem cells can secrete potent combinations of trophic factors, such as cytokines, chemokines, and growth factors, which modulate the molecular composition of the environment to evoke responses from resident cells [38]. Although important, the data presented here also show great promise and suggest the potential of chemotaxis-driven therapy to target exogenous cells to distal anatomical sites such as the skin, emphasizing the need for better cell selection strategies.

The functionality of the CXCR2^+^-mADSC population enriched by exposing cells to TGF-β was further evaluated in a preclinical mouse model of severe DEB. The cells were systemically transplanted into neonatal mice via IP injection. Although intravenous (IV) injection would be more suitable for the systemic transplantation, an extremely high incidence of pulmonary embolism in newborn DEB mice did not allow pursuing the IV route. Nevertheless, IP injection led to a rapid recruitment and accumulation of the CXCR2^+^-mADSC in the blistering skin. Moreover, migrated cells were capable of secreting type VII collagen, with partial reconstitution of the damaged BMZ. Importantly, an estimate of the fluorescence intensity indicated that the type VII collagen level corresponded to about 50 % of the wild-type level. Prior studies demonstrated that 10 % of the BMZ-associated type VII collagen is enough to stabilize the skin of *Col7a1*^*f lNeo/f lNeo*^ hypomorphic mice [29]. Moreover, injection of 20 × 10^6^ fibroblasts resulted in an increase of type VII collagen to approximately 30 % of the wild-type level and provided resistance of the skin against shearing forces [29]. Our group also demonstrated that intradermal transplantation of only 0.5 × 10^6^ congenic MSC led to an increase of type VII collagen to about 15 % of the wild-type level as well as restoration of the damaged DEJ and protection of the skin from mechanical damage [8]. The data presented here demonstrated that greater deposition levels of therapeutic protein could be achieved after systemic transplantation of a rather small number (0.5 × 10^6^cells/injection) of CXCR2^+^-enriched mADSC, emphasizing the great potential of this approach for clinical application. Also, these data are in a good agreement with recent findings showing that preconditioning of MSC with TGF-β, TNF-α, and CXCL12 increases secretion of type VII collagen [39], suggesting that pretreatment of cells with a combination of factors stimulating both the lead receptor and the therapeutic protein may provide better outcome of transplantation therapy.

## Conclusions

In summary, the current study provided experimental proof that the CXCR2^+^-mADSC population possesses blistering skin-homing capabilities allowing targeting of the therapeutic cells directly to DEB-affected skin along a natural chemotactic axis and deposition of type VII collagen into the BMZ with ultimate restoration of the integrity at the DEJ. Currently ongoing in-vitro and in-vivo studies will further refine the procedures for the enrichment of human ADSC with skin-homing capabilities and the elevated secretion of type VII collagen and other proteins necessary for proper assembly of the functional collagen fibers for future allogeneic transplantation to rescue the DEB phenotype in the patients. Further mechanistic studies to identify critical factors involved in chemotactic-based directional migration of the stem cells will hopefully play an important role in designing rational approaches toward increasing the efficiency of targeting the disease site.

## Abbreviations

ASC: Adult stem cells
BMZ: Basement membrane zone
BSA: Bovine serum albumin
CCL: Chemokine (C–C motif) ligand
CCR: Chemokine (C–C motif) receptor
CXCL: Chemokine (C–X–C motif) ligand
CXCR: Chemokine (C–X–C motif) receptor
DEB: Dystrophic epidermolysis bullosa
DEJ: Dermal–epidermal junction
DMEM/F12: Dulbecco’s modified Eagle medium
EDTA: Ethylenediaminetetraacetic acid
FACS: Fluorescence-activated cell sorting
FBS: Fetal bovine serum
H&E: Hematoxylin and eosin
mADSC: Mouse adipose-derived stem cells
MSC: Mesenchymal stem cells
PBS: Phosphate-buffered saline
RT-PCR: Reverse-transcriptase polymerase chain reaction
